# Screening of immune-related differentially expressed genes from primary lymphatic organs of broilers fed with probiotic *bacillus cereus* PAS38 based on suppression subtractive hybridization

**DOI:** 10.1371/journal.pone.0235476

**Published:** 2020-07-01

**Authors:** Jiajun Li, Wanqiang Li, Zhenhua Wang, Abdul Khalique, Junrui Wang, Miao Yang, Xueqin Ni, Dong Zeng, Dongmei Zhang, Yan Zeng, Qihui Luo, Bo Jing, Kangcheng Pan

**Affiliations:** 1 College of Veterinary Medicine, Sichuan Agricultural University, Chengdu, Sichuan Province, China; 2 Branch of Animal Husbandry and Veterinary Medicine, Chengdu Vocational College of Agricultural Science and Technology, Chengdu, Sichuan Province, China; 3 Technology Centre of Chengdu Custom, Chengdu, Sichuan Province, China; University of Illinois, UNITED STATES

## Abstract

To explore the molecular mechanism of the effect of *Bacillus cereus* PAS38 on the immunity of broilers, sixty 7-day-old broilers were divided into two groups with three replicates. The control group was fed with basal diet, and the treatment group was fed with basal diet containing *Bacillus cereus* PAS38 1×10^6^ CFU/g. Thymus and bursa of fabricius were taken from two groups of broilers at the age of 42 days, total RNA was extracted, differential gene library was constructed by SSH technology, and immune-related differential genes were screened. Then, we used siRNA to interfere with the expression of some differential genes in the original generation lymphocytes of broiler blood to detect the change of cytokines mRNA expression level. A total of 42 immune-related differentially expressed genes were screened, including 22 up-regulated genes and 20 down-regulated genes. When 7 differentially up-regulated genes associated with enhanced immune function were interfered with in lymphocytes, some immune-promoting cytokines were down-regulated. These results showed that *Bacillus cereus* PAS38 might up-regulate the expression of *JCHAIN*, *PRDX1*, *CD3E*, *CDK6* and other genes in immune organs of broilers, thereby affecting the development of immune organs, the expression of various cytokines and the transduction of immune signals, improving the immune capacity of broilers.

## Introduction

The most prominent feature of probiotic *Bacillus* is that it can produce stress-resistant spores under adverse environmental conditions such as high temperature, ultraviolet radiation and chemical reagents. It can also tolerate stomach acid and bile salt in animal digestive tract, thus, it has strong stress resistance and environmental adaptability [1]. Various studies have shown that probiotic *Bacillus* preparation can not only promote the growth and development of animal, but also can be used as an immune activator, promote the development of immune organs, activate immune-related signaling pathways, and improve the immune capacity of animals, hence, it is widely used in the poultry industry [2, 3].

*Bacillus cereus* is a common soil bacterium. Some of its strains have been proved to be used as probiotics, and have been developed as probiotics and applied in the field of animal husbandry and veterinary [4]. Zhao et al. [5] reported that adding *Bacillus cereus* EN25 to the diet of sea cucumber could significantly improve the immune function and reduce the cumulative mortality after infection by *Vibrio*. Scharek et al. [6] found that *Bacillus cereus* var. Toyoi could increase the number of CD8+ T cells and γδ T cells in jejunal epithelium and Peyer's lymph nodes of sows and piglets, and reduce the frequency of pathogenic *Escherichia coli* in piglets' feces. Similarly, Roos et al. [7] found that *Bacillus cereus* var. Toyoi as adjuvant could effectively improve the effect of BoHV-5 vaccine, and increase the expression of cytokines such as *IFN-γ*, *IL-12* and *IL-10* in mouse blood. Feng et al. [8] fermented wheat bran with *Bacillus cereus* which could produce xylanase, and fed it to broilers, found that it could improve the amylase activity of duodenal and the abundance of intestinal flora. It was also found that *Bacillus cereus* fed *Carassius auratus gibelio* could significantly ameliorate the immunosuppression, oxidative stress and intestinal flora disorder caused by metal cadmium, and effectively alleviate cadmium poisoning [9, 10].

*Bacillus cereus* PAS38 is a strain isolated from the gut of animal that can produce high levels of cellulase and amylase. Previous studies have shown that the addition of *Bacillus cereus* PAS38 to the diet of broilers can improve the growth performance of broilers, improve the activity of intestinal digestive enzymes, regulate the intestinal microecological environment, promote the development and maturity of immune organs, increase the content of serum immunoglobulin and the level of cytokines such as IRF1 and IL-1 [11, 12]. However, at present, there are few studies on the molecular mechanism of the effect of *Bacillus cereus* PAS38 on broiler immune system, so it is necessary to examine differential expression genes to understand the probiotic mechanism of the strain.

Suppression subtractive hybridization (SSH) technique combines the advantages of suppression PCR and subtractive hybridization, which can enrich rare transcripts with differences and is very suitable for samples with highly similar genetic backgrounds, so it is often used to screen differentially expressed genes in the field of zoology [13–15]. Our group has used SSH to construct the splenic differential gene library of broilers fed *Bacillus cerus* PAS38, and screened 9 immune-related differential genes including *JCHAIN*, *FTH1*, *IGF1R*, *TLR7* and others [16].

In this study, *Bacillus cereus* PAS38 was fed to white feather broiler. Primary lymphoid-organs (thymus and bursa of fabricius) of the broilers were collected at 42 days of age, and total RNA was extracted to synthesize cDNA. Then the differentially expressed gene library was constructed through SSH technology to screen the differentially expressed genes related to immunity. Absolute qRT-PCR was used to verify the differential genes, and siRNA gene interference technology was used to analyze the function of differential genes. So as to preliminarily explore the key genes regulating immunity of *Bacillus cereus* PAS38, and lay a foundation for wide application in broilers production.

## Materials and methods

### Ethics statement

All animal experiments were performed in accordance with the guidelines for the care and use of laboratory animals and approved by the Institutional Animal Care and Use Committee of Sichuan Agricultural University (approval number: DYS20174513-1).

### Laboratory animals and strains

Sixty one-day-old avian white feather broilers were purchased from Sichuan Zhengda Animal and Poultry Co., Ltd. *Bacillus cereus* PAS38 strain was provided by the Animal Microecology Research Center of Sichuan Agricultural University. *Bacillus cereus* PAS38 preparation was obtained by solid fermentation at 37˚C for 48 h. Corn powder was added to regulate the number of viable spores to 10^9^ CFU/g.

### Lymphocytes

Lymphocytes were isolated and purified from peripheral blood of healthy broilers by Animal Microecology Research Center of Sichuan Agricultural University.

### Experimental design

Sixty one-day-old avian white feather broilers were pre-fed with basic diet for 7 days to stabilize the metabolic conditions. The broilers were randomly divided into control group and treatment group, each group consisted of three replicates with 10 chickens per replicate. The control group was fed the basal diet. The treatment group was fed the basal diet supplemented with 0.1% *Bacillus cereus* PAS38 preparation. The experimental broilers were raised in single-layer cage with ten broilers in each cage. The broilers of control group and the treatment group were fed in two animal houses respectively to avoid spore interference. Broilers were fed the diet and water *ad libitum*. At the age of 42 days, two broilers were randomly selected from each replicate of each group. Then the broilers were executed by exsanguination from the carotid artery under anaesthesia with diethylether, and the thymus and bursa of fabricius were taken and immediately stored in liquid nitrogen.

### Construction of SSH libraries

The tissues were ground rapidly in liquid nitrogen, and total RNA was extracted by Trizol reagent. 1.0 μg total RNA was taken and the Smarter^TM^ PCR cDNA Synthesis Kit was used to synthesize double-stranded cDNA. During the synthesis of the second-stranded cDNA, from the 18^th^ cycle to the 33^th^ cycle, 5.0 μL mixture was taken out every 3 cycles for 1.2% agarose gel electrophoresis to select the optimal number of cycles. The double-stranded cDNA was digested by Rsa I enzyme, and the adaptor was connected as the tester, and the unconnected cDNA as the driver. The cDNA in the treatment group was used as tester, and the cDNA in the control group was used as driver for hybridization, which was regarded as SSH forward library; on the contrary, the reverse library used the cDNA in the control group as tester, and the cDNA in the treatment group as driver for hybridization. Then the Advantage 2 PCR Kit was used to conduct nested PCR on the hybridization products to amplify the differential ESTs. Finally, differential ESTs were connected to pBM16A T vector and transformed into competent *Escherichia coli*, which were coated on LB solid medium containing ampicillin antibiotics, IPTG and x-gal, and cultured overnight in a constant temperature incubator. The detailed reaction systems and reaction procedures are described by Li et al. [16].

### Bacterial liquid PCR verification and sequencing

200 white single colonies were randomly selected from each library, and cultured in 1 mL LB liquid medium containing ampicillin antibiotics in a constant temperature incubator overnight. Then, 1.0 μL bacterial liquid was extracted for bacterial liquid PCR to detect whether the differential gene fragment was successfully connected to the vector, and the size and band unicity of the inserted fragment.

The bacterial liquid with positive PCR results was selected and sequenced by Beijing TsingKe Biological Technology Co., Ltd. After the plasmid sequence and nested PCR primer sequence were removed from the sequencing results, the sequencing results were blasted through the blast function of NCBI website (https://www.ncbi.nlm.nih.gov/), and then the repetitive sequences, vector sequences, non mRNA sequences and the sequences that remained unmatched were removed, so as to count the effective sequences.

### Screening and verification of immune-related genes

Through Blast2Go software, GO annotation on Level 2 of SSH library was analyzed, and immune-related genes were screened. Nine genes were selected from the immune-related differential genes of each organ, and absolute qPCR was used to further verify whether they matched the results of SSH. The standard curve was made through the plasmid standard. At the same time, 1 μg single-stranded cDNA was used as template to detect the different genes. The results of qPCR were analyzed by Bio-Rad CFX manager 3.1 software, and the standard curve was obtained to calculate the copies of differential expressed genes. The detailed reaction systems and reaction procedures were described by Li et al. [16]. The primers of differential expressed gene and their optimum annealing temperatures are shown in Table 1.

**Table 1:** Primer sequence of differential genes.

Gene	Forward primer Sequence (5'-3')	Reverse primer Sequence (5'-3')	Annealing temperature (℃)	Product length (bp)
*JCHAIN*	GGTTCGTCCTTGT GGCAGGTTATC	GAGGTCACCGTTA CGCACTTACAC	58	88
*DOCK10*	GGCCACAGCTCAG ATGAAGGAAC	TGAGAGCAGCGAT GTGAATGTAGC	60	191
*BLNK*	TTGGGTTCTGCGG GTAGGGAAG	GGCTGGCACGGCG AGTTTG	60	122
*BTLA*	ACAAGGAGCCGGGTCAGAAGG	AGCTGAGGTGGGC TAGACATGG	63	114
*C7*	GTGCTGCCGTGAG CGACTTC	GCCTGATGCTGACGATGGTGAC	62	102
*CD3E*	AGAGCGAGAGAAGGAGGAGCAC	CACCAACCACACACAGCAGGAG	62	81
*CD74*	CATCCTGGTGGCACTGCTGATC	AGGGTCTGCGAGGTCTTGGTC	62	102
*CDK6*	GCTAAAGCCCACT GCCTAGC	GAGAAGAGTGCGGTGTCTGC	60	92
*IPO7*	CAGCTCAACGAGG CTCATAA	TGCCTGTCTCACTGGTAAATC	64	93
*PRDX1*	CTGCTGGAGTGCG GATTGTG	TGGCATTACAGCTGTGGCAG	62	115
*PTPN6*	GGCCAGTCTCACATTCCTGC	GGCTTGTACAGGCAAGGGAC	62	134
*RAC2*	CAGCCCACCCGAA CGAAGAAAC	AAAGCACAGTGACCGCCAGAAG	58	145
*RPS3*	TCGAGCCTGCTACGGTGTCC	GCACGCTGACCCCTGAGTTTG	55	93
*SATB1*	CAGGGAACACAGCCGAACAGC	CAGGGTGCAGGTTTGGAAGTGG	62	93

### Functional analysis of differential genes

Some genes were selected from the immune-related differential genes verified by qPCR, and their complete mRNA sequences were obtained according to NCBI website. The corresponding siRNA sequences and negative siRNA sequence were designed and synthesized by Shanghai GenePharma Co., Ltd.

The siRNA interference test of lymphocytes was divided into three groups: the treatment group (Group A) was interfered by the siRNA of different genes, the negative control group (Group B) was interfered by the negative siRNA, and the blank control group (Group C) was added with RNAase-free water as control. Each group was divided into three replicates. The experiment was conducted in 24-well plates, each well contained 500 μL lymphocytes (1×10^6^/mL), and the final concentration of siRNA was 30 nM/L. After 48 h of interference, the total RNA of the cells was extracted and reverse transcribed using the M-MLV 4 First-Strand cDNA kit.

Absolute qPCR was used to detect mRNA expression of the interfered gene to verify the interference effect. Finally, the cDNA of lymphocytes with successful interference was selected, and the mRNA expression levels of 6 cytokines: *IL-1B*, *IL-2*, *IL-6*, *MYD88*, *IRF1*, *TLR4* were detected by absolute qPCR to analyze the effect of differential gene silencing on immune gene expression. The primers of 6 cytokines and their optimal annealing temperatures are shown in Table 2.

**Table 2:** Primer sequence of cytokines.

Gene	Forward primer Sequence (5'-3')	Reverse primer Sequence (5'-3')	Annealing temperature (℃)	Product length (bp)
*IL-1B*	CTCGCCTGGATTC TGAGCACAC	GCCTCCGCAGCAGT TTGGTC	63	123
*IL-2*	AAAGTGAGTGTGG GCTTCTC	GCTCTACCTGTATG GCAAGAAT	60	124
*IL-6*	TTCACCGTGTGCG AGAACAGC	CAGCCGTCCTCCTC CGTCAC	61	80
*MYD88*	AAGGTGTCGGAGG ATGGTGGTC	GGAATCAGCCGCTT GAGACGAG	61	120
*IRF1*	CACTAATTGGCCC TCTCTCTTC	GACCTGGGTAAGTG TGCTAAA	60	149
*TLR4*	GCCATCCCAACCC AACCACAG	CCCACTGAGCAGCA CCAATGAG	60	122

### Statistical analysis

All the experimental data were analyzed by one-way ANOVA with SPSS 23.0 software and Duncan’s method was used for multiple comparisons, with P<0.05 as the statistically significant, P<0.01 as the highly statistically significant.

## Results

### Synthesis of double-stranded cDNA

**Fig 1:**
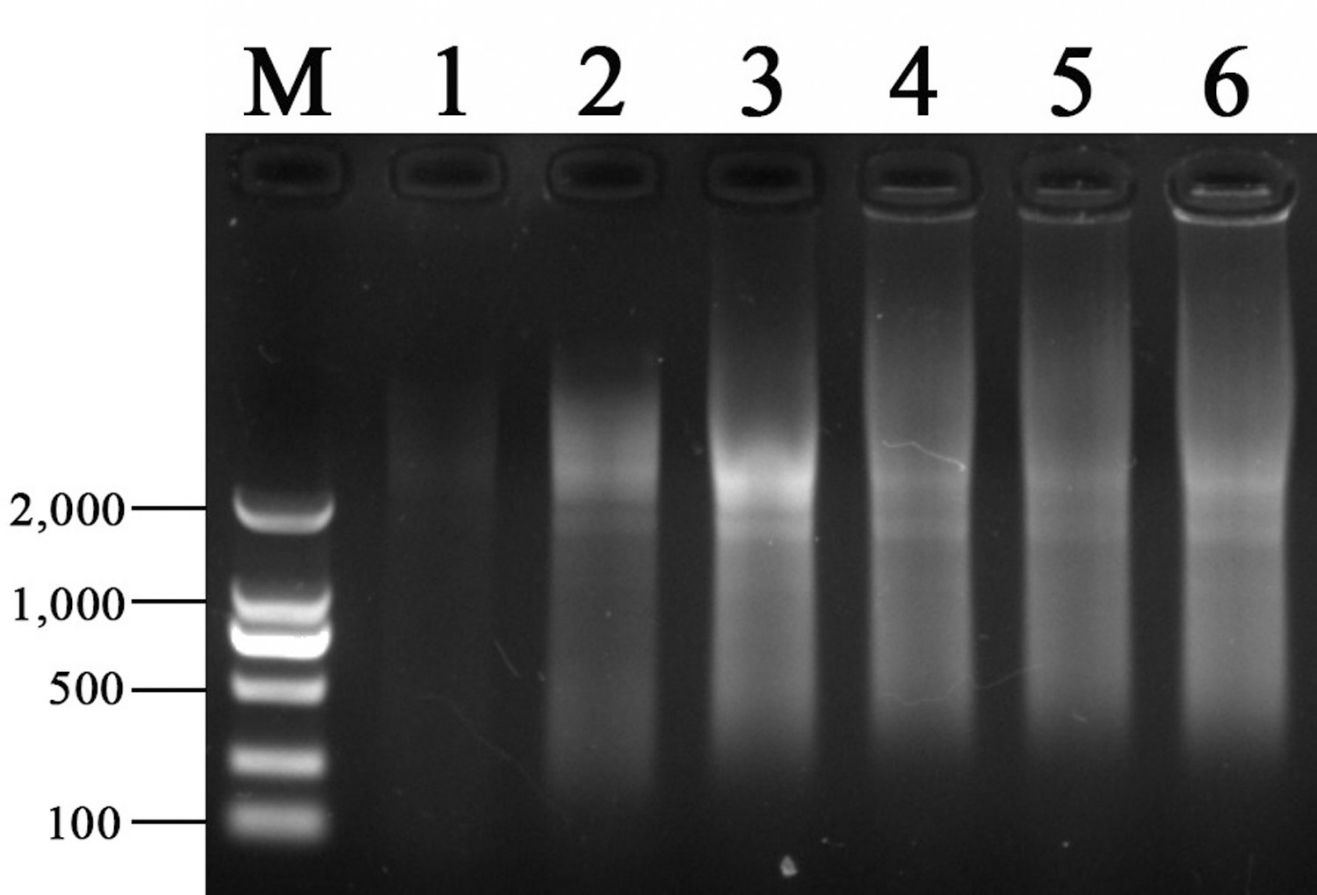
Analysis of optimum PCR cycles number of double-stranded cDNA synthesis. Electrophoresis with agarose of 1.2% concentration. The M represents Marker 2000 (bp), and the lanes 1, 2, 3, 4, 5 and 6 respectively represent the double stranded cDNA products when the PCR cycles are 18, 21, 24, 27, 30 and 33.

Double-stranded cDNA was synthesized by different PCR cycles, and 1.2% agarose gel electrophoresis was shown in Fig 1 (S1 Fig). It can be seen that the brightness and dispersion range of the bands are the best when the cycles are 24. If the number of cycles is more than 24, the brightness of band decreases instead. If the number of cycles is less than 24, and the brightness and range of the bands are significantly lower. In order to better enrich transcripts, especially rare transcriptional information, 24 cycles were selected for synthesis of double-stranded cDNA.

### Detection of bacterial liquid PCR

**Fig 2:**
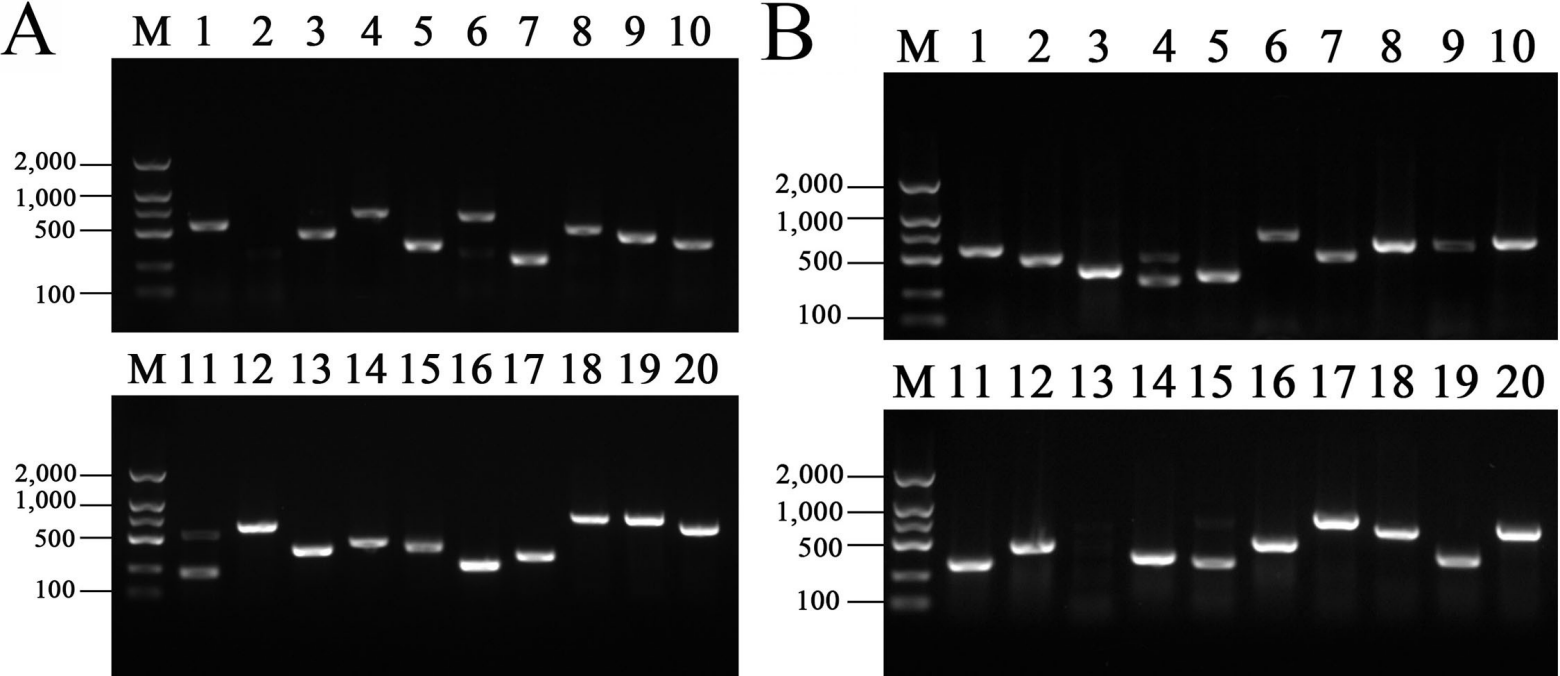
Detection of bacterial liquid PCR. Electrophoresis with agarose of 1.2% concentration. (A) represents thymus; (B) represents bursa of fabricius. M represents marker 2000 (bp). Lanes 1–20 represents PCR products of different bacterial liquid.

The partial results of bacterial liquid PCR are shown in Fig 2 (S2 Fig). It could be seen that the size of bands is concentrated between 200 bp-1000 bp, and most of the bands are single bright bands, and the bacterial liquid with single bright bands is selected for sequencing.

### Sequencing results statistics

The sequencing results were blasted through NCBI, and the valid ESTs were counted, as shown in Table 3. Among them, 128 and 122 differential ESTs were screened separately from the forward and reverse library of the thymus, and 118 and 96 differential ESTs were screened separately from the forward and reverse library of bursa of fabricius. And these ESTs were submitted to NCBI’s dbEST database with the GenBank accession numbers JZ982461-JZ982710 (Thymus) and JZ982751-JZ982964 (Bursa of Fabricius).

**Table 3:** Sequencing results statistics.

Item	Forward library		Reverse Library	
	Thymus	Bursa of fabricius	Thymus	Bursa of fabricius
Successfully sequenced sequences	193	154	194	179
Vector sequences	18	16	20	40
Unmatched sequences	12	14	20	11
Unannotated protein sequences	1	1	0	3
Repeat sequences	34	5	32	29
Effective sequences	128	118	122	96

### GO annotation analysis of SSH library

**Fig 3:**
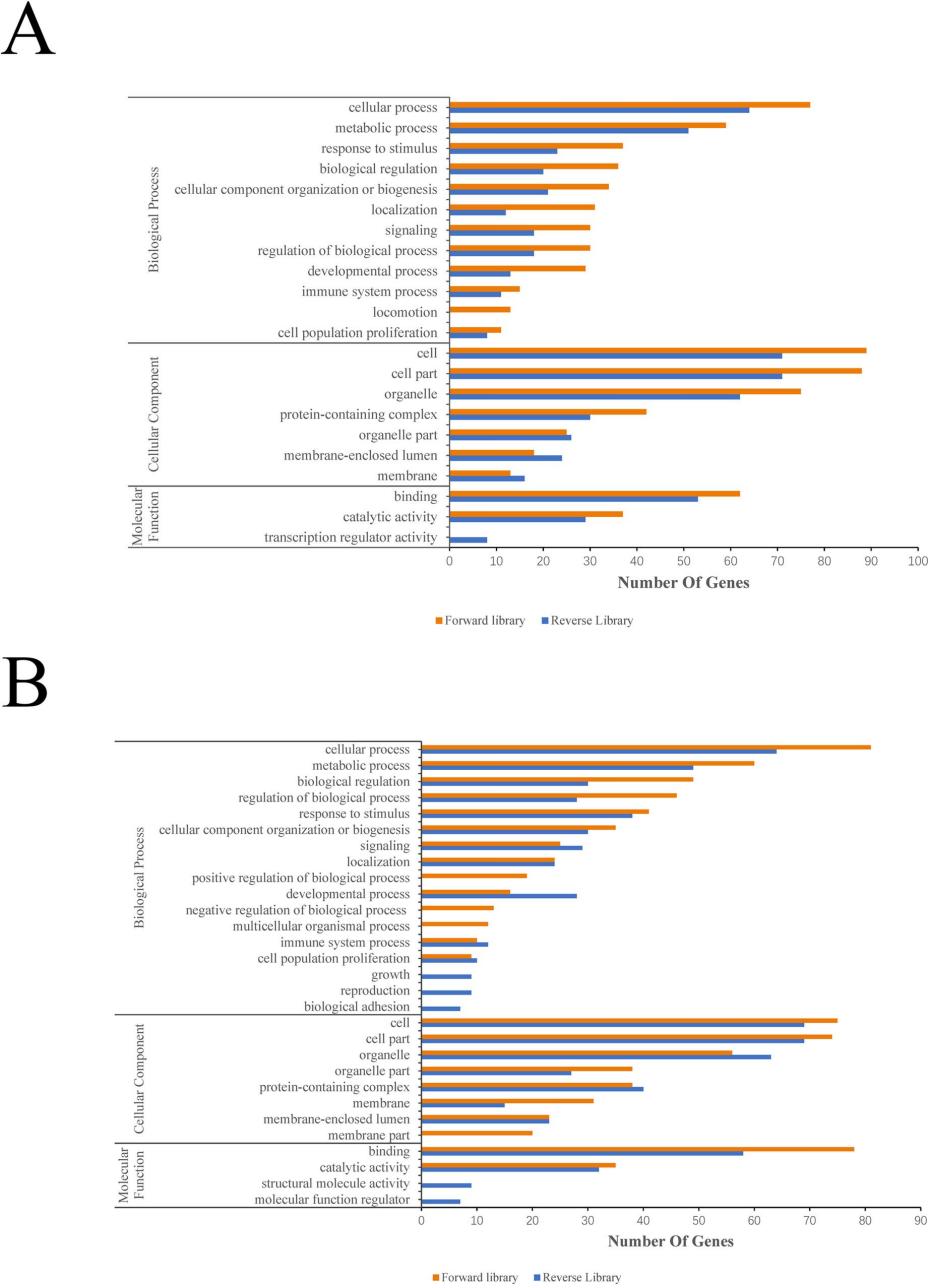
GO annotation of SSH differential genes. (A) represents GO annotation of thymus; (B) represents GO annotation of bursa of fabricius.

Through Blast2Go software, Go annotation analysis of the differential genes in SSH library was performed, and the results are shown in Fig 3. The ESTs of SSH differential gene library in thymus were involved in the biological process including 12 categories, among which the ESTs in forward library were mainly involved in cellular process (19%), metabolic process (15%), response to stimulus (9%), biological regulation (9%), etc., and 4% of ESTs were involved in the immune system process; the ESTs in reverse library were mainly involved in cellular process (25%), metabolic process (20%), response to stimulus (9%), cellular component organization or biogenesis (8%), and 4% of ESTs were also involved in the immune system process. In the classification of cellular component, differential ESTs in thymus includes 7 categories. The forward library was mainly involved in cell (25%), cell part (25%), organelle (21%), protein-containing complex (12%), etc.; the reverse library was mainly involved in cell part (24%), cell (24%), organelle (21%), protein-containing complex (10%), etc. Differential ESTs in thymus were involved in 3 categories of molecular function, with forward library involved in binding (63%) and catalytic activity (37%), the reverse library involved in binding (59%), catalytic activity (32%), and transcription regulator activity (9%) (Fig 3A).

There were 17 categories which the SSH ESTs of bursa of fabricius involved in, the forward library mainly included cellular process (18%), metabolic process (14%), biological regulation (11%), and regulation of biological process (10%), etc., 2% of the ESTs were involved in immune system process; the reverse library was mainly involved in cellular process (17%), metabolic process (13%), response to stimulus (10%), cellular component organization or biogenesis (8%), etc., and 3% of ESTs were involved in immune system process. In the classification of cellular component, there were 8 categories which the differential ESTs of bursa of fabricius involved in, the forward library was mainly involved in cell (21%), cell part (21%), organelle (16%), organelle part (11%), etc.; the reverse library was mainly involved in cell part (23%), cell (23%), organelle (21%), protein-containing complex (13%), etc. There were 4 categories of molecular function involved in the differential ESTs of bursa of fabricius, including binding (69%) and catalytic activity (31%) in the forward library; and binding (55%), catalytic activity (30%), structural molecular activity (8%), and molecular function regulator (7%) in the reverse library (Fig 3B).

### Screening and verification of immune-related differential genes

Through the GO annotation, immune-related differential genes were screened separately from SSH libraries of two organs, as shown in Table 4. There were 26 immune-related genes in thymus, 15 of which were up-regulated and 11 down-regulated. There were 21 immune-related differentially expressed genes in bursa of fabricius, 10 of which were up-regulated and 11 down-regulated.

**Table 4:** Screening of immune-related differential genes.

Organ	Forward library (Up-regulate)		Reverse Library (Down-regulate)	
	Gene	Accession number	Gene	Accession number
Thymus	*STAT1*	XM_025152161.1	*FYB*	XM_025144741.1
	*PRDX1*	NM_001271932.1	*RAB7A*	XM_025154624.1
	*DHRS7B*	XM_015294395.2	*RPS3*	NM_001030836.1
	*JCHAIN*	NM_204263.1	*DDX60*	XM_015285390.2
	*MAD2L2*	NM_001025578.1	*SATB1*	XM_025147207.1
	*CDK6*	NM_001007892.3	*BLNK*	XM_015288574.2
	*YPEL5*	XM_015278080.2	*RTKN2*	XM_025151545.1
	*RTN4*	NM_001348287.2	*IPO7*	XM_003641394.4
	*TMOD3*	NM_001005813.1	*PAG1*	XM_004939933.3
	*RAC2*	NM_001201452.1	*SP3*	XM_025152088.1
	*CD3E*	NM_206904.1	*PRKAR1A*	XM_015279894.2
	*BCL2*	XM_015854617.1		NM_001030836.1
	*CCR6*	XM_015284122.2		
	*LYST*	XM_015284416.2		
	*PTPRC*	XM_015290239.2		
Bursa of Fabricius	*C7*	NM_001318402.1	*AIMP1*	XM_420496.6
	*HPRT1*	NM_204848.1	*PTPN6*	XM_015293521.2
	*MSN*	NM_001031112.1	*RIF1*	XM_025152555.1
	*FAS*	XM_015288353.2	*ELF1*	NM_001006269.1
	*FAU*	XM_025145444.1	*IPO7*	XM_003641394.4
	*BTLA*	XM_429579.6	*RPS14*	NM_001030619.1
	*JCHAIN*	NM_204263.1	*RPS3*	NM_001030836.1
	*SPI1*	XM_015286975.2	*CD74*	XM_015293754.2
	*CDK6*	NM_001007892.3	*KLHL6*	NM_001031303.2
	*PRDX1*	NM_001271932.1	*PPP6C*	NM_001079765.1
			*RHOA*	XM_025154420.1

**Fig 4:**
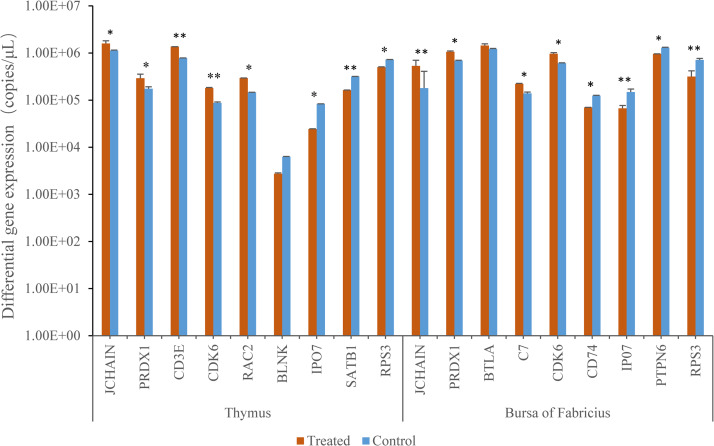
The expressions of immune-related differential genes were confirmed by absolute qPCR. In the figure, “*” above the column indicates significant difference (P<0.05), while “**” indicates extremely significant difference (P<0.01).

Absolute qPCR was performed separately on 9 genes selected from the immune-related differentially expressed genes of two organs to verify whether they matched the results of SSH. The copies of differential genes were calculated by the standard curve (S3A–S3M Fig). The results showed that the expression of 9 genes (5 up-regulated genes, 4 down-regulated genes) in thymus had significant difference except for the down-regulated gene *BLNK*, but the copies of *BLNK* in the treatment group was also down 57% compared with that in the control group. The expressions of 9 genes in bursa of fabricius (5 up-regulated genes, 4 down-regulated genes) were also significantly different except for the up-regulated gene *BTLA*, the copies of *BTLA* in the treatment group was increased by 19% compared with that in the control group. This shows that the detection results of qPCR were basically consistent with screening results of SSH (Fig 4).

### Functional analysis of differential genes

Considering the relationship between primary lymphoid organs and secondary lymphoid organs, and the results of our study on spleen differential genes [16], we selected these seven differential genes: *JCHAIN*, *FTH1*, *IGF1R*, *PRDX1*, *CD3E*, *TLR7* and *CDK6* to further analyze their gene functions. Shanghai GenePharma Co., Ltd. was entrusted to design and synthesize corresponding siRNA and negative control siRNA, and the siRNA sequence is shown in Table 5.

**Table 5:** siRNA sequence of immune-related differential gene.

Gene	Forward sequence (5'-3')	Reverse sequence (5'-3')
JCHAIN-1004	CCACGGACAUCAGCAGAAATT	UUUCUGCUGAUGUCCGUGGTT
FTH1-253	GCCUUUGCUUUGGCGUGUATT	UACACGCCAAAGCAAAGGCTT
IGF1R-2865	CCUCAGCAUGUCCUACUAUTT	AUAGUAGGACAUGCUGAGGTT
PRDX1-564	CCACGUCAUUGGCUGGAAATT	UUUCCAGCCAAUGACGUGGTT
CD3E-343	CCUUCCAGUUUACAGAUAATT	UUAUCUGUAAACUGGAAGGTT
TLR7-1624	GCAGGUGAUCAGGAACAUATT	UAUGUUCCUGAUCACCUGCTT
CDK6-781	GCUGAUUCCUACCAACCUUTT	AAGGUUGGUAGGAAUCAGCTT
Negative Control	UUCUCCGAACGUGUCACGUTT	ACGUGACACGUUCGGAGAATT

The interference results of 7 immune-related differential genes are shown in Table 6. It could be seen that, compared with the negative siRNA control (Group B) and the blank control (Group C), the copies of the 7 differential genes after the corresponding siRNA interference (Group A) were significantly reduced (P<0.01), and there was no significant difference between the negative siRNA control group and the blank control group (P>0.05).

**Table 6:** Detection of interference effect of different genes siRNA (copies/μL). Different lowercase on the upper right of the same row of data indicates significant difference (P<0.05), different capital indicates highly significant difference (P<0.01), and the same letter or no mark indicates no significant difference (P>0.05).

Differential	Group		
Gene	A	B	C
*JCHAIN*	297.00±85.61^B^	692.00±27.71^A^	725.33±44.60^A^
*FTH1*	248859.00±34246.11^B^	766096.00±62250.42^A^	741834.33±34153.52^A^
*IGF1R*	6263.00±168.01^B^	26307.33±605.51^A^	23726.67±2772.88^A^
*PRDX1*	4645.33±224.91^B^	20994.67±2477.08^A^	21734.67±1252.17^A^
*CD3E*	13049.67±1592.65^B^	33153.67±1988.76^A^	31918.33±2219.06^A^
*TLR7*	878.00±31.10^B^	2312.33±160.86^A^	2662.33±304.59^A^
*CDK6*	1556.33±309.41^B^	6397.67±889.76^A^	6243.00±752.35^A^

The cDNA of lymphocytes after the interference of differential genes was taken, and the mRNA expression levels of 6 cytokines were detected by absolute qPCR (S4A–S4F Fig), as shown in Table 7. The expressions of *IL-1B*, *IL-2*, *IL-6* and *TLR4* were highly significantly down-regulated after interference of *JCHAIN* and *PRDX1* (P<0.01). After *FTH1* was interfered, *IL-1B*, *IL-2*, *IL-6*, *TLR4* and *MYD88* were significantly down-regulated (P<0.01). After the interference of *IGF1R*, cytokines *TLR4*, *IL-6*, *MYD88* and *IL-1B* were significantly down-regulated (P<0.01). After *TLR7* was interfered in lymphocytes, *IL-6* and *IL-1B* were significantly down-regulated (P<0.01). After interfering with the expression of *CDK6* and *CD3E*, the copies of all six cytokines were significantly reduced (P<0.05).

**Table 7:** Effect of siRNA interference on cytokines expression (copies/μL). Different lowercase on the upper right of the same row of data indicates significant difference (P<0.05), different capital indicates highly significant difference (P<0.01), and the same letter or no mark indicates no significant difference (P>0.05).

Differential Gene	Cytokine	Group		
		A	B	C
*JCHAIN*	*IRF1*	48966.33±7273.51^b^	63454.00±12873.45^a^	52833.67±7664.13^ab^
	*TLR4*	993.00±51.96^B^	6833.33±651.83^A^	6974.33±246.38^A^
	*IL-6*	1479.33±34.50^B^	4351.67±508.94^A^	4914.33±505.66^A^
	*MYD88*	5153.33±636.24	4985.67±849.12	4877.67±886.68
	*IL-1B*	8720.33±523.31^B^	49402.33±2666.20^A^	47071.67±6487.62^A^
	*IL-2*	5619.00±1474.92^B^	13900.67±3730.55^A^	12781.00±4010.25^A^
*FTH1*	*IRF1*	50392.00±9706.58	63454.00±12873.45	52833.67±7664.13
	*TLR4*	1446.00±57.16^B^	6833.33±651.83^A^	6974.33±246.38^A^
	*IL-6*	2125.33±494.90^B^	4351.67±508.94^A^	4914.33±505.66^A^
	*MYD88*	967.00±186.20^B^	4985.67±849.12^A^	4877.67±886.68^A^
	*IL-1B*	27790.67±4450.76^B^	49402.33±2666.20^A^	47071.67±6487.62^A^
	*IL-2*	5292.00±813.67^B^	13900.67±3730.55^A^	12781.00±4010.25^A^
*IGF1R*	*IRF1*	43437.00±7883.42^B^	63454.00±12873.45^A^	52833.67±7664.13^AB^
	*TLR4*	2451.67±336.58^B^	6833.33±651.83^A^	6974.33±246.38^A^
	*IL-6*	3499.67±243.13^B^	4351.67±508.94^A^	4914.33±505.66^A^
	*MYD88*	2239.33±351.14^B^	4985.67±849.12^A^	4877.67±886.68^A^
	*IL-1B*	30733.33±2468.88^B^	49402.33±2666.20^A^	47071.67±6487.62^A^
	*IL-2*	12206.33±1833.89	13900.67±3730.55	12781.00±4010.25
*PRDX1*	*IRF1*	63327.33±11472.68	63454.00±12873.45	52833.67±7664.13
	*TLR4*	1996.00±448.66^B^	6833.33±651.83^A^	6974.33±246.38^A^
	*IL-6*	2634.33±181.54^B^	4351.67±508.94^A^	4914.33±505.66^A^
	*MYD88*	4442.33±356.64	4985.67±849.12	4877.67±886.68
	*IL-1B*	22225.33±777.22^B^	49402.33±2666.20^A^	47071.67±6487.62^A^
	*IL-2*	6919.67±159.51^B^	13900.67±3730.55^A^	12781.00±4010.25^A^
*CD3E*	*IRF1*	33491.33±3446.20^B^	63454.00±12873.45^A^	52833.67±7664.13^A^
	*TLR4*	1012.33±378.72^B^	6833.33±651.83^A^	6974.33±246.38^A^
	*IL-6*	1298.67±45.94^B^	4351.67±508.94^A^	4914.33±505.66^A^
	*MYD88*	1925.33±326.83^B^	4985.67±849.12^A^	4877.67±886.68^A^
	*IL-1B*	9784.33±587.01^B^	49402.33±2666.20^A^	47071.67±6487.62^A^
	*IL-2*	6534.00±1821.60^B^	13900.67±3730.55^A^	12781.00±4010.25^A^
*TLR7*	*IRF1*	52983.00±3992.81	63454.00±12873.45	52833.67±7664.13
	*TLR4*	6314.33±290.52^b^	6833.33±651.83^ab^	6974.33±246.38^a^
	*IL-6*	2155.33±76.49^B^	4351.67±508.94^A^	4914.33±505.66^A^
	*MYD88*	4640.67±697.12	4985.67±849.12	4877.67±886.68
	*IL-1B*	17294.00±1564.04^B^	49402.33±2666.20^A^	47071.67±6487.62^A^
	*IL-2*	12720.67±2160.35	13900.67±3730.55	12781.00±4010.25
*CDK6*	*IRF1*	36444.00±3902.65^Bb^	63454.00±12873.45^A^	52833.67±7664.13^ABa^
	*TLR4*	1270.00±74.12^B^	6833.33±651.83^A^	6974.33±246.38^A^
	*IL-6*	3046.67±177.51^B^	4351.67±508.94^A^	4914.33±505.66^A^
	*MYD88*	1601.00±136.43^B^	4985.67±849.12^A^	4877.67±886.68^A^
	*IL-1B*	20366.33±4121.13^B^	49402.33±2666.20^A^	47071.67±6487.62^A^
	*IL-2*	8858.33±629.43^b^	13900.67±3730.55^a^	12781.00±4010.25^a^

## Discussion

In this study, SSH technology was used to construct the differential gene libraries of thymus and bursa of fabricius in broilers fed with *Bacillus cereus* PAS38. Through sequencing, sequence alignment and statistics, the SSH library of thymus contains 250 ESTs, 51.2% of which were up-regulated and 48.8% were down regulated. There were 214 ESTs in the SSH library of bursa of fabricius, 55.14% of which were up-regulated and 44.86% were down regulated. At the same time, the GO annotation of these libraries showed that most of the genes were mainly involved in biological process or molecular function such as cellular process, metabolic process, catalytic activity, binding, etc., and some genes were also involved in the immune system process. This indicates that the molecular mechanism of *Bacillus cereus* PAS38 affecting the physiological activities of broilers is comprehensive and complex, which might regulate the growth, development, metabolism and immunity of broilers through multiple signaling pathways.

According to the GO annotation of SSH library, 42 immune-related differential expression genes were screened from two organs, 54.76% of them were up-regulated and 45.24% down regulated. Nine immune-related differentially expressed genes were selected from each of the two organs for absolute qPCR verification, and the results showed that the expression level of 88.89% genes was significantly changed compared with the control group. Although the changes in the expression levels of *BLNK* and *BTLA* were not significant, they also changed to some extent, which was basically consistent with the screening results of SSH, indicating that SSH technology is generally reliable and can be used to isolate differential genes.

Some of these immune-related differential genes were differentially expressed in both thymus and bursa of fabricius. Among them, *JCHAIN*, *PRDX1* and *CDK6* were significantly up-regulated in both organs, while *IPO7* and *RPS3* were significantly down-regulated. In our previous study, *JCHAIN* was also significantly up-regulated in the spleen [16]. JCHAIN protein (Joining chain of multimeric IgA and IgM) is a glycoprotein that can initiate polymerization by linking to homocysteine in the tail of IgM and IgA and bind to poly IgM and IgA stably by isulfide bonds, and *JCHAIN* plays an important role in the secretion and transport of immunoglobulin, and the activation of complement [17, 18]. JCHAIN protein has highly conserved structural characteristics in various vertebrates and invertebrates. It has been found that the amino acid composition of JCHAIN protein in chicken and human is highly similar [19]. Some scholars have studied the expression level of *JCHAIN* in adult chicken brain, intestine, thymus, spleen, bursa of fabricius and other tissues and organs, and found that the expression level in spleen and rectum was higher, while the expression level in thymus is lower [20]. In addition, some studies have shown that *Lactobacillus bulgaricus* can activate lymphocytes, synthesize IgA dimer through *JCHAIN*, and significantly increase the level of sIgA in breast milk and feces of lactating women [21]. In this experiment, the expression of *JCHAIN* in the immune organs of broilers increased significantly after feeding *Bacillus cereus*, which may be a positive signal for the immunity of broilers.

*PRDX1* (Peroxiredoxins 1) is a member of the *PRDXs* (peroxiredoxins) family. It can balance the H_2_O_2_ level which is very important for signal transmission and metabolism through catalytic oxidation-reduction. Therefore, *PRDX1* may be a good regulator of "H_2_O_2_" signal [22]. Matulova et al. [23] found that *PRDX1* was up-regulated in the spleen of broilers infected with *Salmonella enteritidis*. Lavrič et al. [24] also found that the expression of *PRDX1* increased after macrophages of chicken were exposed to Mycoplasma synovium or *Escherichia coli*. In addition, some studies have shown that the expression of PRDX1 protein in kidney of broilers decreased after infectious bronchitis coronavirus infection [25]. These two different results suggest that chickens may have different defense mechanisms in response to bacterial and viral infection, and *PRDX1* may play a role in chicken resistance to bacterial infection. In this study, *PRDX1* gene was up-regulated in thymus and bursa of fabricius of broiler chickens fed with probiotics. Thymus and bursa of fabricius are important places for the body to produce lymphocytes. It suggests that *Bacillus cereus* PAS38 may promote lymphocytes production of *PRDX1* in immune organs, so as to improve the ability of organism to deal with bacterial infection.

*CDK6* (Cyclin-dependent kinases 6) is a cell cycle regulator, and the activity of CDK6 protein depends on its binding to Cyclins. It can combine with Cyclin D to form a complex, thus transform the cell cycle from G1 to S [26, 27]. Uras et al. [28] found that the lack of *CDK6* would make red blood cells more sensitive to mechanical stress in vitro and lead to a shorter life span, which would lead to anemia in mice. Grison et al. [29] found that *CDK6* deficiency could slow the proliferation of basal progenitor cells in the dorsal and ventral forebrain of mice. These studies indicate that *CDK6* plays an important role in the development of organism. Meanwhile, Henrique et al. [30] found that the high expression of *CDK6* in the cytoplasm of CD8 memory cells was conducive to the rapid cell division, which showed that *CDK6* also played a role in the immune process. In this experiment, the expression of *CDK6* in thymus and bursa of fabricius increased after broilers were fed with *Bacillus cereus*. Combined with previous studies, *Bacillus cereus* can promote the development of immune organs and increase the number of lymphocyte [6, 11], indicating that *Bacillus cereus* PAS38 may achieve these effects by up regulating *CDK6* gene.

*IPO7* (Importin-7), as a member of the nuclear adaptor protein importin-β superfamily, is a cell transporter, which can mediate proteins, hormones and nucleic acids into the nucleus [31]. At present, the research on *IPO7* is mainly focused on its transport mechanism of various proteins and hormones in organism, and there are few studies on immunity [32]. However, some researchers have found that HIV-1 virus can use *IPO7* to maximize the nuclear input of its DNA genome [33]. Others have found that the expression level of *IPO7* is related to the clinical symptoms of human asthma [34]. After feeding broilers with *Bacillus cereus* PAS38, the expression of *IPO7* in thymus and bursa of fabricius decreased, which might be beneficial to inhibit the proliferation of virus in vivo.

*RPS3* (Ribosomal protein S3) belongs to ribosomal protein S3P family and is a part of ribosomal 40s subunit [35]. *RPS3* has endonuclease activity and is believed to play a role in repairing damaged DNA and apoptosis [36, 37]. *RPS3* leads kappa light chain enhancer of NF-κB subunit of activated B cells to specific κB site, which plays an important role in the innate response of bacterial infection. Wu et al. [38] found that *E*. *coli* had multiple mechanisms to block *RPS3* mediated transcriptional activation, thus interfering with innate immunity. Park et al. [39] found that RPS3 could also act as a binding protein of TLR4 to induce the maturation and activation of dendritic cells, which has a potential auxiliary role in dendritic cell tumor vaccine. In this experiment, after the broilers were fed with *Bacillus cereus* PAS38, the *RPS3* expression in thymus and bursa of fabricius was down-regulated in the treatment group, which might be related to the reduction of anaphylaxis. Of course, its specific mechanism of action needs further study.

In this experiment, 6 cytokines were selected to detect the expression changes after the interference of differential genes. Among them, *IL-1B*, *IL-2* and *IL-6* are all common interleukins, they play an important role in lymphocytes activation, production of cytokines, antibodies and complement [40–42]. And *MYD88*, *IRF1* and *TLR4*, play key roles in pathogen recognition, activation and conduction of immune signals [43–45].

The expression of *IL-1B*, *IL-2*, *IL-6* and *TLR4* in the lymphocyte decreased significantly after *JCHAIN* was interfered. These three kinds of interleukin and *TLR4* are mainly produced by mature macrophages and T cells. In previous studies, *JCHAIN* was mainly related to the secretion of immunoglobulin [18]. This experiment shows that in broilers, *JCHAIN* may also have influence on the generation of certain interleukins and toll-like receptors, and participate in the process of immune activation. Similarly, interference with *PRDX1* in lymphocytes also resulted in significant down-regulation of *IL-1B*, *IL-2*, *IL-6*, and *TLR4*. PRDX1 is an important signaling protein that can regulate intracellular H_2_O_2_ level. Combined with the results of this experiment, it is suggested that *PRDX1* may play a role in the resistance of chickens to bacterial infection through the activation of immune signals.

*FTH1* (Ferritin heavy chain 1) is the main subunit of ferritin, responsible for the oxidation and integration of ferrous ions to maintain the balance of ferrous ions in the body [46]. Some studies have shown that *FTH1* is related to the egg-laying traits of chickens [47], and some studies have also proven that expression of *FTH1* changed significantly after poultry were infected with some pathogenic bacteria and viruses [23, 48]. After the interference of *FTH1*, the five cytokines: *IL-1B*, *IL-2*, *IL-6*, *TLR4* and *MYD88* were all significantly down-regulated. This suggests that in broilers, *FTH1* may be involved in the defense mechanism against viruses and bacteria by influencing the production of some cytokines.

*IGF1R* (Insulin Like Growth Factor 1 Receptor) can combine with insulin like growth factor (IGFs) specifically to mediate IGFs signaling and promote the development of various tissues and organs [49]. Previous studies have reported that *IGF1R* played a positive role in weight gain and bone growth and development in broilers [50], and selenium deficiency in broilers could inactivate the IGF-1R/PI3K/Akt/mTOR pathway, reduce the growth rate of the spleen and the number of splenic lymphocytes [51]. This study found that, after the interference of *IGF1R*, the expression of *TLR4*, *IL-6*, *MYD88* and *IL-1B* were significantly down-regulated, which suggests that *IGF1R* may regulate the expression of certain cytokines by affecting the development of immune organs and the number of lymphocytes in broilers, play a positive regulatory role in the development of the immune system.

When *TLR7* (Toll-like receptor 7) was down-regulated by interference in lymphocytes, the copies of *IL-6* and *IL-1B* were significantly reduced. *TLR7* belongs to the toll-like receptor family and is mainly expressed in the lungs and spleen, secreted by macrophages, B lymphocytes and mast cells [52]. It can be activated by single strand RNA, and then activates NF-κB pathway through MyD88 and TRAF6, it can lead to activation, leading to cytokines secretion and inflammatory response [53]. It has been found that the levels of *IFN-β*, *IFN-γ*, *IL-1β* and *IL-4* in peripheral blood mononuclear cells of broilers were significantly up-regulated after *TLR7* agonist Resiquimod was used [54]. It has also been found that the highly virulent NDV strain can block the expression of *TLR7* in chicken macrophages and enhance replication [55]. In this study, interference of *TLR7* down-regulated two kinds of interleukins, suggesting that *TLR7* may be involved in the defense against viruses by promoting the expression of immune factors in broilers.

After interfering with the expression of *CDK6* and *CD3E*, the copies of all six cytokines were significantly reduced. *CDK6* is a cell cycle regulator that plays a role in the development and the immune system of organism [26–30]. CD3E (Cluster of differentiation 3e) is a one-way type I membrane glycoprotein located on the surface of T cells [56]. Mainly as one of the subunits of T cell receptor CD3 complex, it plays an important role in antigen recognition, various intracellular signal transduction and T cell development, and also initiates the assembly of CD3 complex [57]. At present, the research on *CDK6* and *CD3E* in broiler immunity has not been reported. This experiment should be the first time to explore its immune function in broilers. It was found that the interference of these two genes led to the down-regulation of six cytokines. It is speculated that *CDK6* may participate in the proliferation process of lymphocytes, and *CD3E* may participate in the development of T cells and intracellular signal transduction, thus affecting the expression of many cytokines.

Through interference tests on these 7 differential genes, it was preliminarily analyzed that the mechanism of *Bacillus cereus* PAS38 to improve broiler immunity might be as follows: (1) By up-regulating the expression of some growth factors, such as *IGF1R* and *CDK6*, it can promote the development of immune organs and the proliferation of lymphocytes. (2) By promoting the expression of *JCHAIN*, *PRDX1* and *FTH1*, it is beneficial to the production of immunoglobulin, and interleukin, toll like receptor and other cytokines. (3) By up-regulating genes such as *TLR7* and *CD3E* that play important functions in antigen recognition and intracellular signal transduction, which can affect the expression of various cytokines and also promote the development of immune cells, so as to enable the body to cope with the invasion of bacteria and viruses more efficiently.

## Conclusion

The expression of many genes in thymus and bursa of fabricius were significantly affected after broilers were fed with *Bacillus cereus* PAS38, including 42 immune-related genes. When some of the immune-related differential genes were interfered in the original generation lymphocytes of broiler, some cytokines were significantly down-regulated. It is suggested that *Bacillus cereus* PAS38 may promote the development of immune organs and the proliferation of lymphocytes by up-regulating some genes of immune organs such as *JCHAIN*, *PRDX1*, *CDK6*, and *CD3E*, etc., which is conducive to the activation and transmission of immune signals, as well as the synthesis of cytokines and immunoglobulins, thus play a regulatory role in the immune system of broilers.

## Supporting information

S1 Fig Agarose electrophoresis analysis of optimal cycles of double-stranded cDNA. Electrophoresis with agarose of 1.2% concentration. The images were generated by the Gel imaging system Gel Doc™ XR+. The M represents Marker 2000 (bp), and the lanes 1, 2, 3, 4, 5 and 6 respectively represent the double stranded cDNA products when the PCR cycles are 18, 21, 24, 27, 30 and 33.
(TIF)

S2 Fig Detection of bacterial liquid PCR. Electrophoresis with agarose of 1.2% concentration. The images were generated by the Gel imaging system Gel Doc™ XR+. (A) represents thymus; (B) represents bursa of fabricius. M represents marker 2000 (bp). Lanes 1–20 represents PCR products of different bacterial liquid. Fig 2A was generated by S2A Fig, and Fig 2B was generated by S2B Fig.
(TIF)

S3 Fig Absolute qPCR standard curve of differential genes in thymus and bursa of fabricius. A-L represents respectively differential gene *JCHAIN*, *PRDX1*, *CD3E*, *CDK6*, *RAC2*, *BLNK*, *IPO7*, *SATB1*, *RPS3*, *BTLA*, *C7*, *CD74*, *PTPN6*.The abscissa represents the concentration of plasmid standard (Log_10_N copies/μL). The longitudinal coordinates denote the cycle threshold.
(TIF)

S4 Fig Absolute qPCR standard curve of cytokines. A-F represents respectively cytokines *IRF1*, *TLR4*, *IL-6*, *MYD88*, *IL-1B*, *IL-2*. The abscissa represents the concentration of plasmid standard (Log_10_N copies/μL). The longitudinal coordinates denote the cycle threshold.
(TIF)

S1 Table Raw material composition and nutritional level of basic dietary (air-dry basis). Premix is provided for feed per kg: VD3 200 IU, VA 1500 IU, VE 10 IU, VK 0.5 mg, VB12 0.01 mg, VB6 3.0 mg, VB1 1.5 mg, Nicotinic acid 30 mg, D-pantothenic acid 10 mg, Folic acid 0.5 mg, Biotin 0.15 mg, Trace elements Cu, Fe, Zn, Mn, Se, I are 8 mg, 80 mg, 40 mg, 60 mg, 0.15 mg, 0.18 mg respectively. Metabolic energy was calculated and the rest was measured.
(PDF)
