# Supplementary figures and images for: Screening of immune-related differentially expressed genes from primary lymphatic organs of broilers fed with probiotic bacillus cereus PAS38 based on suppression subtractive hybridization
Source: PLoS One. 2020 Jul 1;15(7):e0235476. doi: 10.1371/journal.pone.0235476 (PMC7329121; doi:10.1371/journal.pone.0235476)

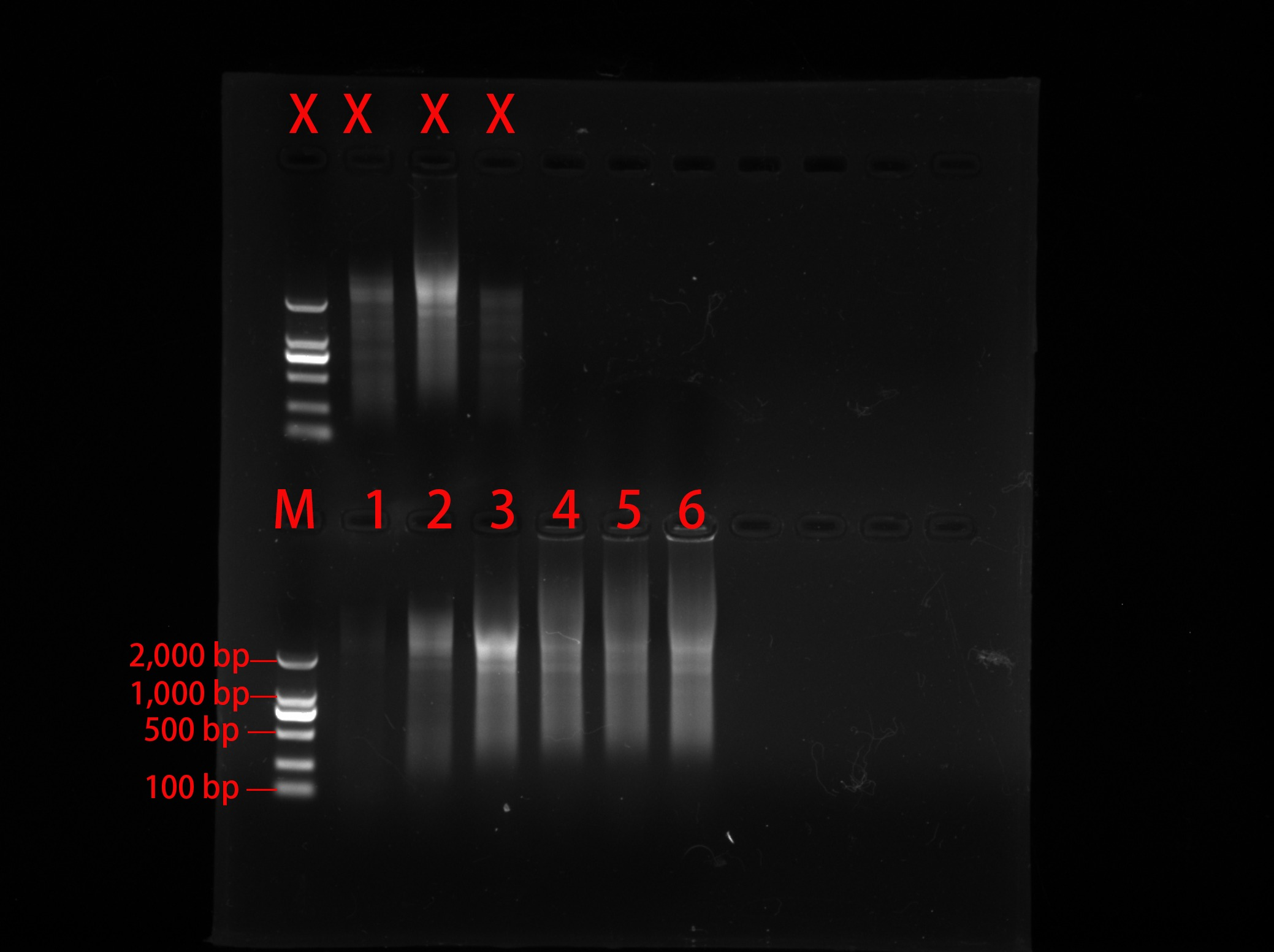

Supplement: S1 Fig — Electrophoresis with agarose of 1.2% concentration. The images were generated by the Gel imaging system Gel Doc™ XR+. The M represents Marker 2000 (bp), and the lanes 1, 2, 3, 4, 5 and 6 respectively represent the double stranded cDNA products when the PCR cycles are 18, 21, 24, 27, 30 and 33. (TIF) [file pone.0235476.s001.tif]

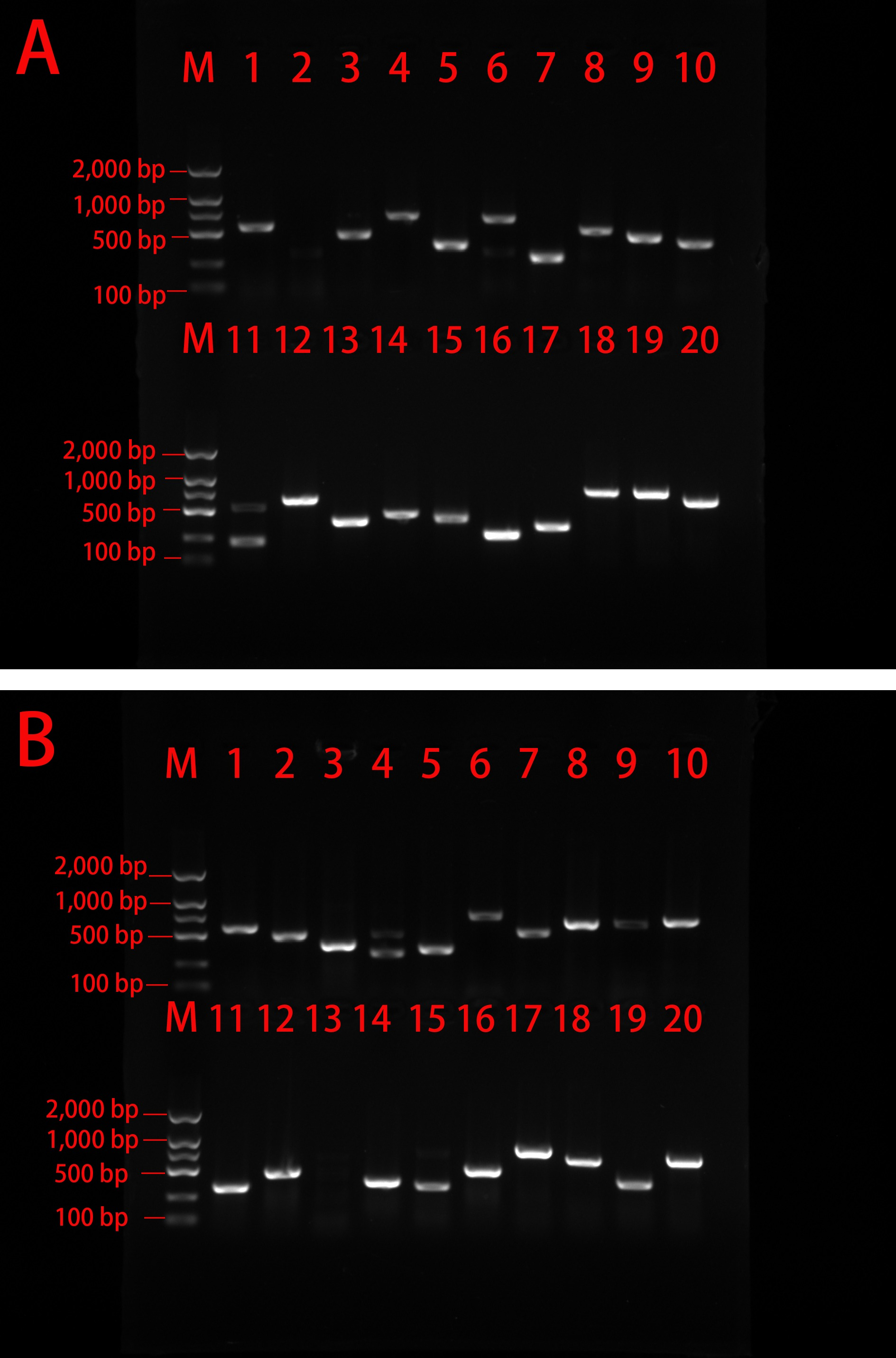

Supplement: S2 Fig — Electrophoresis with agarose of 1.2% concentration. The images were generated by the Gel imaging system Gel Doc™ XR+. (A) represents thymus; (B) represents bursa of fabricius. M represents marker 2000 (bp). Lanes 1–20 represents PCR products of different bacterial liquid. Fig 2A was generated by S2A Fig, and Fig 2B was generated by S2B Fig. (TIF) [file pone.0235476.s002.tif]

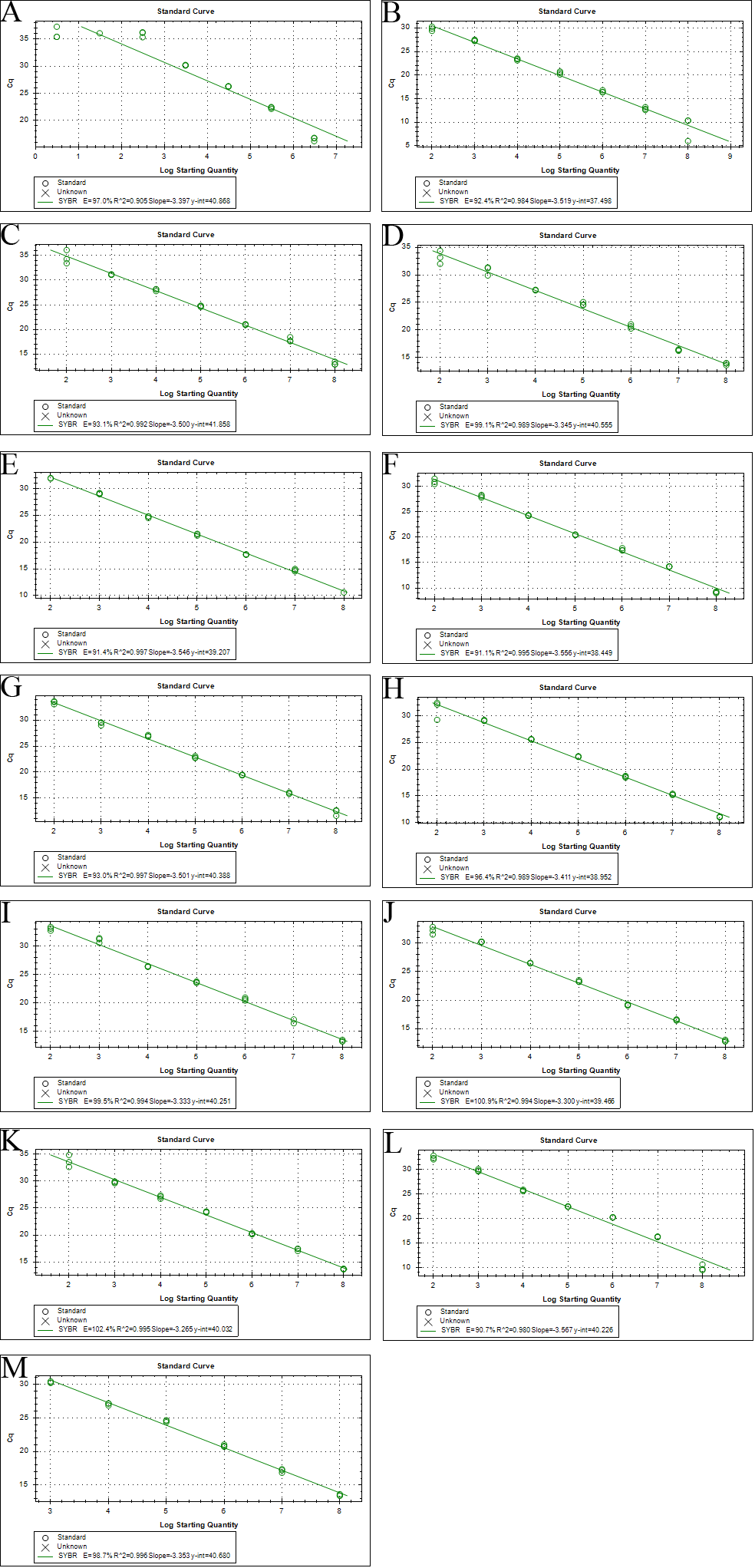

Supplement: S3 Fig — A-L represents respectively differential gene JCHAIN, PRDX1, CD3E, CDK6, RAC2, BLNK, IPO7, SATB1, RPS3, BTLA, C7, CD74, PTPN6.The abscissa represents the concentration of plasmid standard (Log10N copies/μL). The longitudinal coordinates denote the cycle threshold. (TIF) [file pone.0235476.s003.tif]

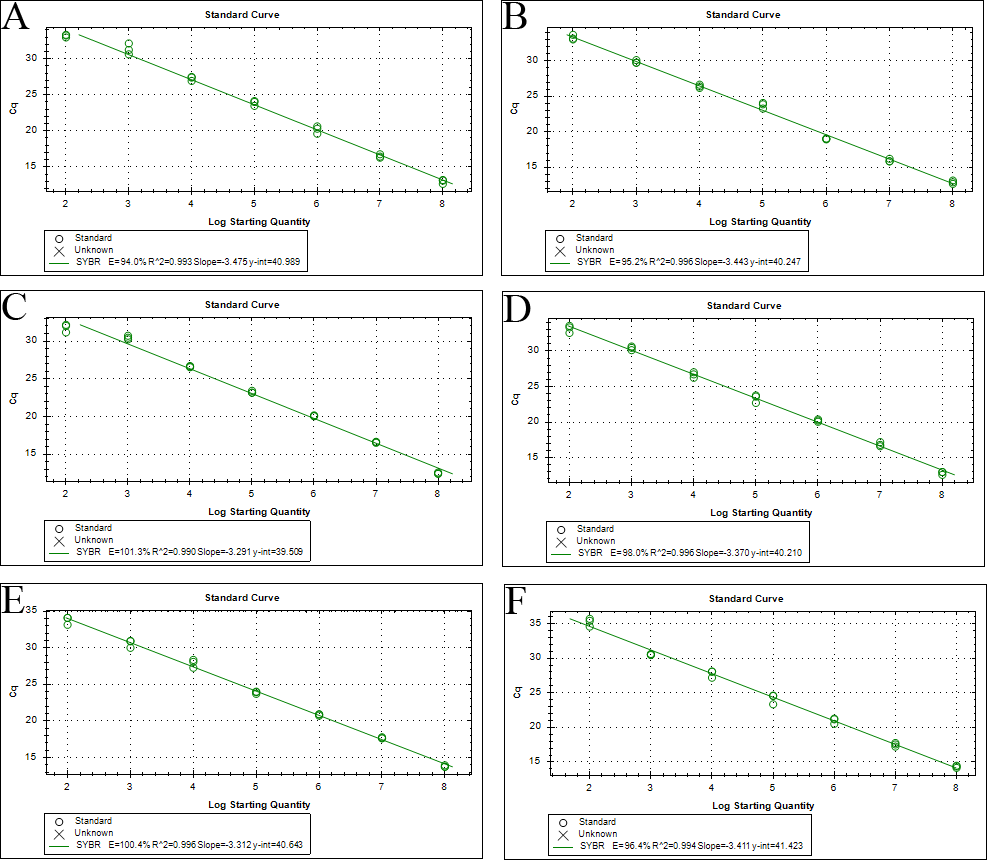

Supplement: S4 Fig — A-F represents respectively cytokines IRF1, TLR4, IL-6, MYD88, IL-1B, IL-2. The abscissa represents the concentration of plasmid standard (Log10N copies/μL). The longitudinal coordinates denote the cycle threshold. (TIF) [file pone.0235476.s004.tif]
