# Supplementary material for: Screening of immune-related differentially expressed genes from primary lymphatic organs of broilers fed with probiotic bacillus cereus PAS38 based on suppression subtractive hybridization
Source: PLoS One. 2020 Jul 1;15(7):e0235476. doi: 10.1371/journal.pone.0235476 (PMC7329121; doi:10.1371/journal.pone.0235476)
Supplement: S1 Table — Premix is provided for feed per kg: VD3 200 IU, VA 1500 IU, VE 10 IU, VK 0.5 mg, VB12 0.01 mg, VB6 3.0 mg, VB1 1.5 mg, Nicotinic acid 30 mg, D-pantothenic acid 10 mg, Folic acid 0.5 mg, Biotin 0.15 mg, Trace elements Cu, Fe, Zn, Mn, Se, I are 8 mg, 80 mg, 40 mg, 60 mg, 0.15 mg, 0.18 mg respectively. Metabolic energy was calculated and the rest was measured. (PDF) [file pone.0235476.s005.pdf]

Ingredients(%)			Nutrition levels		
	1d-21d	22d-42d		1d-21d	22d-42d
Corn	61.20	65.20	ME/(MJ/kg)	12.54	12.80
Soybean meal	23.00	18.00	CP	20.70	19.00
Extruded soybean	8.50	10.00	Lys	1.12	0.96
Import fish meal	3.00	3.00	Met	0.53	0.43
$CaHPO_4$	1.60	1.40	Ca	0.99	0.89
Limestone	1.10	1.00	AP	0.51	0.46
NaCl	0.32	0.30			
DL-Met	0.18	0.10			
L-Lys	0.10				
Premix	1.00	1.00			
Total	100.00	100.00			
